# The dosimetric error due to uncorrected tumor rotation during real‐time adaptive prostate stereotactic body radiation therapy

**DOI:** 10.1002/mp.16094

**Published:** 2022-11-28

**Authors:** Chandrima Sengupta, Simon Skouboe, Thomas Ravkilde, Per Rugaard Poulsen, Doan Trang Nguyen, Peter B. Greer, Trevor Moodie, Nicholas Hardcastle, Amy J. Hayden, Sandra Turner, Shankar Siva, Keen‐Hun Tai, Jarad Martin, Jeremy T. Booth, Ricky O'Brien, Paul J. Keall

**Affiliations:** ^1^ ACRF Image X Institute University of Sydney Sydney New South Wales Australia; ^2^ Danish Center for Particle Therapy Aarhus University Hospital Aarhus Denmark; ^3^ Department of Oncology Aarhus University Hospital Aarhus Denmark; ^4^ Department of Radiation Oncology Calvary Mater Newcastle Waratah New South Wales Australia; ^5^ Crown Princess Mary Cancer Center Sydney New South Wales Australia; ^6^ Peter MacCallum Cancer Center Melbourne Victoria Australia; ^7^ Sir Peter MacCallum Department of Oncology University of Melbourne Melbourne Victoria Australia; ^8^ Northern Sydney Cancer Center Royal North Shore Hospital Sydney New South Wales Australia

**Keywords:** motion management, motion‐induced dose error, tumor motion

## Abstract

**Background:**

During prostate stereotactic body radiation therapy (SBRT), prostate tumor translational motion may deteriorate the planned dose distribution. Most of the major advances in motion management to date have focused on correcting this one aspect of the tumor motion, translation. However, large prostate rotation up to 30° has been measured. As the technological innovation evolves toward delivering increasingly precise radiotherapy, it is important to quantify the clinical benefit of translational and rotational motion correction over translational motion correction alone.

**Purpose:**

The purpose of this work was to quantify the dosimetric impact of intrafractional dynamic rotation of the prostate measured with a six degrees‐of‐freedom tumor motion monitoring technology.

**Methods:**

The delivered dose was reconstructed including (a) translational and rotational motion and (b) only translational motion of the tumor for 32 prostate cancer patients recruited on a 5‐fraction prostate SBRT clinical trial. Patients on the trial received 7.25 Gy in a treatment fraction. A 5 mm clinical target volume (CTV) to planning target volume (PTV) margin was applied in all directions except the posterior direction where a 3 mm expansion was used. Prostate intrafractional translational motion was managed using a gating strategy, and any translation above the gating threshold was corrected by applying an equivalent couch shift. The residual translational motion is denoted as $T_{res}$. Prostate intrafractional rotational motion $R_{uncorr}$ was recorded but not corrected. The dose differences from the planned dose due to $T_{res}$ + $R_{uncorr}$, ΔD($T_{res}$ + $R_{uncorr}$) and due to $T_{res}$ alone, ΔD($T_{res}$), were then determined for CTV D98, PTV D95, bladder V6Gy, and rectum V6Gy. The residual dose error due to uncorrected rotation, $R_{uncorr}$ was then quantified: $\Delta D_{Residual}$ = ΔD($T_{res}$ + $R_{uncorr}$) ‐ ΔD($T_{\mathrm{res}}$).

**Results:**

Fractional data analysis shows that the dose differences from the plan (both ΔD($T_{res}$ + $R_{uncorr}$) and ΔD($T_{res}$)) for CTV D98 was less than 5% in all treatment fractions. ΔD($T_{res}$ + $R_{uncorr}$) was larger than 5% in one fraction for PTV D95, in one fraction for bladder V6Gy, and in five fractions for rectum V6Gy. Uncorrected rotation, $R_{uncorr}$ induced residual dose error, $\Delta D_{Residual}$, resulted in less dose to CTV and PTV in 43% and 59% treatment fractions, respectively, and more dose to bladder and rectum in 51% and 53% treatment fractions, respectively. The cumulative dose over five fractions, ∑D($T_{res}$ + $R_{uncorr}$) and ∑D($T_{res}$), was always within 5% of the planned dose for all four structures for every patient.

**Conclusions:**

The dosimetric impact of tumor rotation on a large prostate cancer patient cohort was quantified in this study. These results suggest that the standard 3–5 mm CTV‐PTV margin was sufficient to account for the intrafraction prostate rotation observed for this cohort of patients, provided an appropriate gating threshold was applied to correct for translational motion. Residual dose errors due to uncorrected prostate rotation were small in magnitude, which may be corrected using different treatment adaptation strategies to further improve the dosimetric accuracy.

## 1 INTRODUCTION

In prostate stereotactic body radiation therapy (SBRT), high radiation dose is delivered in one or few fractions,^1^ requiring the treatment delivery to be highly accurate and precise. In the continued quest for precise radiation therapy to improve treatment outcomes, methods to detect and correct for intrafraction target translational motion (3 degrees‐of‐freedom, 3 DoFs) have been developed^2^, ^3^, ^4^, ^5^, ^6^, ^7^, ^8^, ^9^, ^10^, ^11^, ^12^, ^13^, ^14^ and increasingly adopted clinically.^4^, ^10^, ^14^ The majority of the innovations in real‐time image‐guided radiation therapy to date have only focused on this one aspect of tumor motion, translation. However, along with translation, large intrafraction prostate rotation has also been observed.^15^, ^16^, ^17^, ^18^ It has been identified that, ideally, both translation and rotation (6 DoF) should be monitored in real‐time during a treatment. As the technological complexity increases when accounting for tumor translation and rotation, an important question is to estimate the clinical benefit that the translational and rotational motion correction has over translational motion correction alone.

**FIGURE 1:**
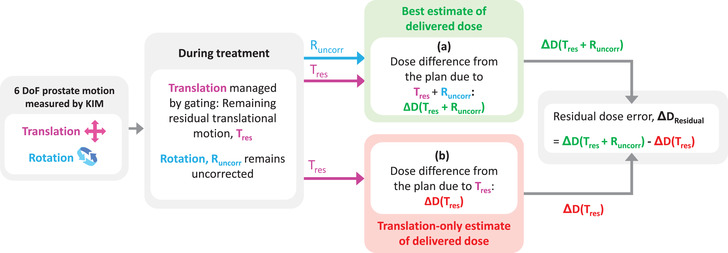
Overview of the study methodology. During treatment, a real‐time tumor motion monitoring system, KIM^18^ provided intrafraction prostate tumor translational and rotational motion. Tumor translational motion was managed using a gating strategy and the residual translation is denoted as $T_{res}$. Tumor rotational motion $R_{uncorr}$ remained uncorrected. Dose reconstruction was performed including (a) $T_{res}$ + $R_{uncorr}$ and (b) only $T_{res}$ and the corresponding dose differences were determined: ΔD($T_{res}$ + $R_{uncorr}$) and $\Delta D(T_{res})$, respectively. The residual dose error due to uncorrected rotation was quantified as: $\Delta D_{Residual}$ = ΔD($T_{res}$ + $R_{uncorr}$) ‐ $\Delta D(T_{res}$).

While the impact of uncorrected intrafraction prostate translation on dose is well addressed in the literature,^19^ the dosimetric impact of uncorrected intrafraction prostate rotation is not well understood. Several studies quantified the dose error arising due to uncorrected prostate rotation.^15^, ^17^, ^20^ Common to all these studies is that the impact of rotational motion on the delivered dose was estimated by applying mean rotation to the structures from their planned positions. Therefore, the interplay effects between dynamic target rotation and the linear accelerator during a treatment fraction were ignored in these studies. Recent studies revealed that dose reconstructed using dynamic rotation could be significantly different to doses reconstructed with a mean rotation.^21^, ^22^ To quantify the impact of prostate rotation on the dynamic delivered dose in a treatment fraction, we reconstructed the delivered dose including dynamic 6 DoF prostate motion for 32 prostate cancer patients treated on a 5‐fraction prostate SBRT clinical trial. This was the best estimate of the delivered dose in this study, which was then compared with the translation‐only (3 DoF) estimate of the delivered dose. The study methodology is demonstrated in Figure 1.

## 2 METHOD

### 2.1 Determination of residual dose error due to uncorrected rotation

To reconstruct the motion‐induced dynamic delivered dose for a treatment fraction, an in‐house developed software named DoseTracker was used that can estimate the delivered dose in the presence of translation and rotation of the tumor.^21^, ^23^, ^24^, ^25^, ^26^ To compute dose within DoseTracker, the clinical treatment plan, structure set and dose distribution were exported as DICOM‐RT files from the treatment planning system (Eclipse, Varian Medical Systems, Palo Alto) and imported into DoseTracker for every patient. DoseTracker uses structure sets to read the patient geometry and assign calculation points. To estimate dose, DoseTracker first calculates the dose distribution in the isocenter plane as a convolution between the time‐averaged field aperture in the time period and a two‐dimensional dose kernel, and then scales the isocenter plane dose to the depths of the calculation points by percentage depth dose curves and the inverse square law.^23^, ^24^ Dose calculation points inside the selected structures are automatically set up in the same dose grid as used by the TPS (defined in the DICOM‐RT dose file).

Motion is included in the dose reconstruction algorithm by shifting each individual calculation point between consecutive dose increment calculations. For motion‐induced dose calculation, DoseTracker shifts all calculation points inside the body contour according to a motion monitoring signal and calculates the dose in each point. If the planned position is *r* = (*x*, *y*, *z*), after rotating and translating, the point is moved to $r^{\prime }$, given by,

(1) $$r^{\prime }=R\left(r-r_{0}\right)+r_{0}+T,$$

where, *r*_0_ = center of rotation, *T* is the translation vector and *R* is the rotation matrix. DoseTracker currently assumes water density within the patient contour. DoseTracker's accuracy in determining the motion‐induced dose error has been demonstrated in studies for both translational and rotational motion.^24^, ^26^ Similar uncertainty is expected for the results presented in this study.

In our study, intrafractional tumor translational and rotational motion were recorded during treatment at 10 Hz. Intrafractional translational motion was managed with a gating strategy and any motion above the gating threshold was corrected by applying an equivalent couch shift. The residual translational motion is denoted as $T_{res}$. Intrafractional tumor rotational motion was not corrected and is denoted as $R_{uncorr}$. To calculate dynamic delivered dose D($T_{res}$ + $R_{uncorr}$) including residual translation and uncorrected rotation, the tumor motion traces, $T_{res}$ and $R_{uncorr}$ and the planned accelerator parameters were sent to DoseTracker (Figure 1, label (a)) as a User Datagram Protocol (UDP) signal at 10 Hz. To compute the dynamic delivered dose, D($T_{res}$) including only residual translation, $T_{res}$ and the planned accelerator parameters were sent to DoseTracker as a UDP signal at 10 Hz (Figure 1, label (b)). DoseTracker reconstructed the motion‐induced and the planned dose increment to all calculation points in a continuous loop. The motion‐induced dose increment was calculated using the accelerator and tumor positions in the UDP messages received since the previous dose increment calculation. The planned dose, $D_{Plan}$, increment was calculated by assuming no tumor motion and using the planned accelerator motion during the actual MU increment as looked up in the DICOM‐RT plan. The median computation time for each dose calculation of DoseTracker typically ranged from 110–320 ms. If data covering more than 500 ms had accumulated in the data queue, the queue was emptied in portions of maximum 500 ms to prevent averaging over longer time intervals. When dose calculation was completed for each treatment fraction, the planned and motion‐induced dose dicoms were exported as DICOM‐RT files to calculate the relevant dose metric for this study.

The motion‐induced dose cubes and the patient structure set from each fraction were imported into 3D Slicer (version 4.11.2)^27^ to export the dose volume histograms. Using 3D Slicer, dose summation was performed over all five fractions to estimate the motion‐induced dose (∑D($T_{res}$ + $R_{uncorr}$) and ∑D($T_{res}$)) for every patient.

The dose differences from the planned dose (a) due to $T_{res}$ + $R_{uncorr}$, ΔD($T_{res}$ + $R_{uncorr}$) and (b) due to $T_{res}$, ΔD($T_{res}$) were determined for each fraction, as well as, for every patient summed over all five fractions.

The dose metric for reporting doses in the presence of motion is not explicitly detailed in International Commission on Radiation Units and Measurements Report 83.^28^ The dose values reported in this work are the clinical target volume (CTV) D98, planning target volume (PTV) D95, and two organs‐at‐risk, bladder V6Gy and rectum V6Gy.

For CTV and PTV, the dose difference equations are:

(2) $$\Delta D\left(T_{res}+R_{uncorr}\right)\left(\%\right)=\frac{(D\left(T_{res}+R_{uncorr}\right)-D_{Plan})\times 100}{D_{Plan}},$$

(3) $$\Delta D\left(T_{res}\right)\left(\%\right)=\frac{(D\left(T_{res}\right)-\;D_{Plan})\times 100}{D_{Plan}}.$$

For bladder and rectum, the dose difference equations are:

(4) $$\begin{array}{ccc} \Delta D\left(T_{res}+R_{uncorr}\right)\left(\%\right) & = & V_{6Gy}(D\left(T_{res}+R_{uncorr}\right))\%\\ & - & V_{6Gy}(D_{Plan})\%, \end{array}$$

(5) $$\Delta D\left(T_{res}\right)\left(\%\right)=V_{6Gy}\left(D\left(T_{res}\right)\right)\%-V_{6Gy}\left(D_{Plan}\right)\%.$$

The rotation of the prostate may not be exactly the same as for the two organs‐at‐risk; however, the assumption in this work is that the high‐dose regions in the bladder and the rectum rotate simultaneously along with the prostate tumor volume.

The residual dose error, $\Delta D_{Residual}$, arising due to the uncorrected dynamic rotation, $R_{uncorr}$, is defined as:

(6) $$\Delta D_{Residual}=\Delta D\left(T_{res}+R_{uncorr}\right)-\Delta D\left(T_{res}\right).$$

$\Delta D_{Residual}$ is the dose error in the estimated impact of motion when only translation rather than the full 6 DoF motion is included in the dose reconstruction.

### 2.2 Planning and treatment

A total of 32 patients were investigated in this study which consisted of 160 fractions treated on the TROG 15.01 SPARK trial (NCT02397317, registered on 24/03/2015) at three centers.^29^ Two fractions were excluded from this study due to insufficient data available, which resulted in a total of 158 fractions. Patients received 7.25 Gy in each fraction using volumetric modulated arc therapy (VMAT) over two arcs with treatment delivered on Varian TrueBeam linacs. A 5 mm CTV to PTV expansion in all directions was used except the posterior direction where a 3 mm expansion was used. The treatment planning dose–volume constraints are given in the SPARK study protocol,^29^ and the relevant dose parameters have been added in Supporting Information in Table S1. The sphericity^30^ of the CTV and the PTV was calculated for all patients, and the results show high sphericity values for both CTV and PTV with low variability. The mean and standard deviation of sphericity of the CTV were 0.91 and 0.07 and that of the PTV were 0.93 and 0.06, respectively, computed for all patients.

Three cylindrical gold fiducial markers (1 mm diameter and 3 mm length) were implanted in the prostate for every patient. The fiducial markers were used for pre‐treatment image matching and intrafraction 6 DoF tumor motion monitoring with kilovoltage intrafraction monitoring (KIM).^18^

### 2.3 Intrafraction tumor motion monitoring

The KIM method converts 2D positions from the kilovoltage x‐ray system at 10 Hz into a real‐time 3D position of the tumor during radiotherapy treatment. The 3D marker position was reconstructed by use of a 3D Gaussian probability density function (PDF),^31^ which was estimated prior to treatment from the setup CBCT projections. During treatment, the PDF was updated with the new‐incoming kV images. The centroid position of the prostate was determined by the average of the three marker positions. The rotational motion was calculated from the 3D marker position using an iterative closest point algorithm.^32^ We have performed extensive analysis to show that the accuracy and precision of the KIM system in determining prostate tumor motion was 0.0±0.5, 0.0±0.4, and 0.1±0.3 mm for translation and −0.1$\pm 0.6^{\circ }$, −0.1$\pm 1.4^{\circ }$, and −0.1$\pm 1.0^{\circ }$ for rotation in the AP, LR, and SI directions, respectively.^18^

While the SPARK study guidelines recommended a 2 mm/5 s KIM gating tolerance, sites participated in the study followed their own protocols. At one center, the KIM gating criteria was set for any motion of > 3 mm persisting for > 5 s in any axis (5 patients). For the other two centers, the KIM gating criteria was set for any motion of > 2 mm persisting for > 5 s in any axis (27 patients).

### 2.4 Observed intrafraction tumor motion

**FIGURE 2:**
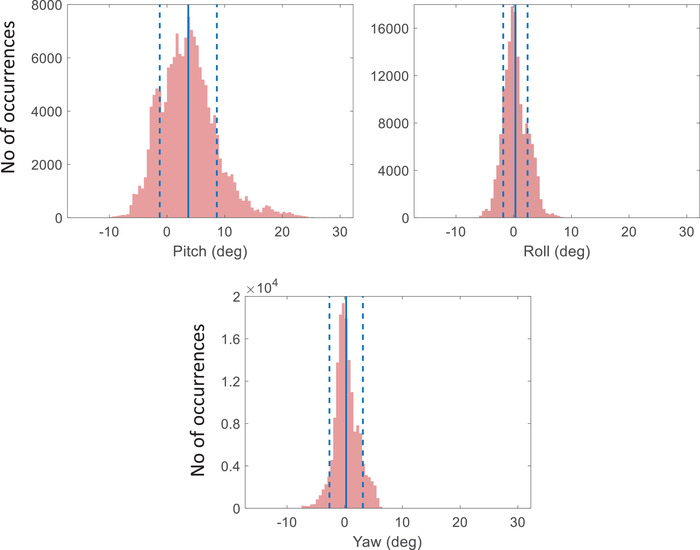
Number of occurrences of rotation $R_{uncorr}$ in pitch, roll, and yaw axes during 158 treatment fractions analyzed in this work. The full blue line shows the mean rotation angle and the dashed lines show 1 standard deviation for each histogram.

The mean, standard deviation, minimum, and maximum of residual tumor translational motion, $T_{res}$, in left‐right (LR), superior‐inferior (SI), and anterior‐posterior (AP) axes for 158 treatment fractions were calculated and the results are given in Table 1. The mean, standard deviation, minimum, and maximum of rotation, $R_{uncorr}$ in pitch, roll, and yaw axes for these treatment fractions are given in Table 2. The histogram in Figure 2 graphically represents the number of occurrences of rotation, $R_{uncorr}$ in each of the three axes. The data show a systematic rotation in the pitch axis with an overall shift of ∼ 4° and extreme rotations up to ∼ 26° can be seen. For both roll and yaw axes, this systematic shift in mean rotation is absent as the rotation frequency distributions for these directions are peaked around ∼ 0° with a smaller variation of rotation angles.

**TABLE 1:** The mean, standard deviation, minimum, and maximum of residual translational motion $T_{res}$ in the three translation axes, LR, SI, and AP, during 158 treatment fractions analyzed in this work.

Translation axis	Mean (mm)	Std. dev. (mm)	Min (mm)	Max (mm)
LR	0.0	0.6	−3.2	3.1
SI	−0.1	1.1	−3.6	3.8
AP	−0.4	0.9	−3.9	3.9

**TABLE 2:** The mean, standard deviation, minimum, and maximum angle of rotation $R_{uncorr}$ in three rotation axes, pitch, roll, and yaw, during 158 treatment fractions analyzed in this work.

Rotation axis	Mean (°)	Std. dev. (°)	Min (°)	Max (°)
Pitch	3.7	4.9	−12.0	26.1
Roll	0.3	2.1	−9.7	9.8
Yaw	0.3	2.1	−9.9	9.1

## 3 RESULTS

**FIGURE 3:**
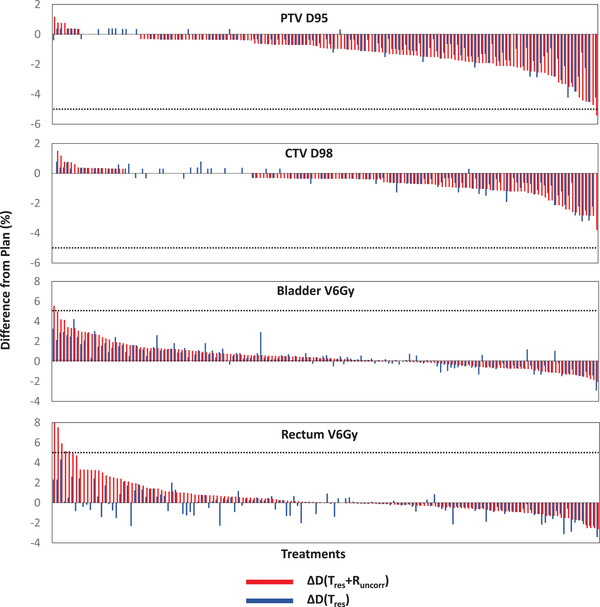
Dose differences from the plan (Equations (2)–(5)) due to $T_{res}$ + $R_{uncorr}$ (ΔD($T_{res}$ + $R_{uncorr}$)) and due to $T_{res}$ (ΔD($T_{res}$)) for all 158 treatment fractions analyzed in this work. The dashed lines indicate a 5% dose difference to planned dose.

Following the method described in Section 2.1, ΔD($T_{res}$ + $R_{uncorr}$) and ΔD($T_{res}$) were calculated using Equations (2)–(5). A positive dose difference indicates more dose to an organ compared to the plan, and, a negative dose difference indicates less dose delivered compared to the planned dose. These dose differences are shown in Figure 3 for all 158 fractions included in this study.

**FIGURE 4:**
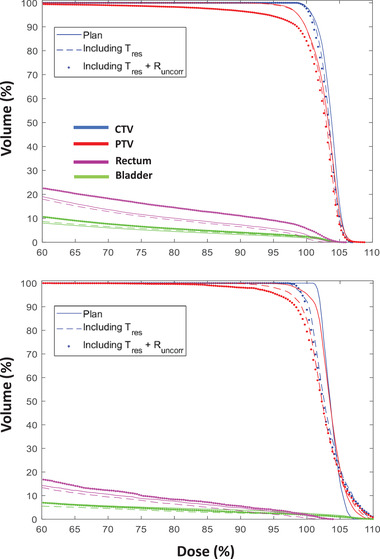
Dose volume histograms for two treatment fractions from two patients where 4.9% (top panel) and 4.5% (bottom panel) underdose occurred in PTV D95 when $T_{res}$ + $R_{uncorr}$ were included in the dose reconstruction. Including $T_{res}$ alone in the dose reconstruction method only shows 0.3% (top panel) and 2.5% (bottom panel) underdose to the PTV, respectively.

To put the results into context, a 5% dose difference between the planned dose and the dose delivered to the patient has been considered clinically meaningful.^33^ In all 158 treatment fractions, ΔD($T_{res}$) was always below 5% for the CTV, PTV, and organs‐at‐risk. When ΔD($T_{res}$ + $R_{uncorr}$) was calculated, > 5% underdosing was seen for PTV D95 in one fraction, > 5% overdosing was seen for bladder V6Gy in one fraction, and for rectum V6Gy in five fractions out of 158 fractions. Figure 4 shows the dose volume histograms of two treatment fractions from two patients where 4.9% and 4.5% underdosing of the PTV D95 occurred when $T_{res}$ + $R_{uncorr}$ were included in the dose reconstruction. Including residual translation $T_{res}$ alone in the dose reconstruction method only shows 0.3% and 2.5% underdose to the PTV, respectively.

The cumulative dose, ∑D($T_{res}$ + $R_{uncorr}$) and ∑D($T_{res}$) for every patient was always within 5% of the planned dose for all four structures.

**FIGURE 5:**
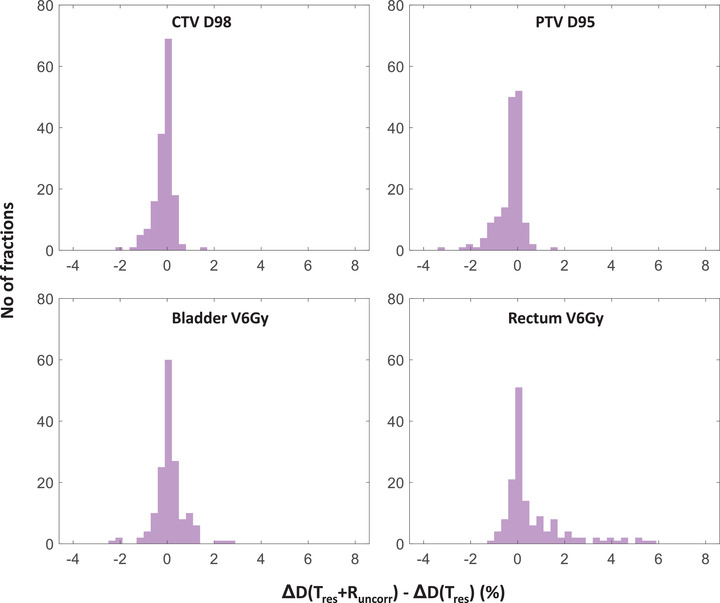
Histogram of the residual dose error $\Delta D_{Residual}$ (Equation (6)) arising due to uncorrected dynamic rotation. The residual dose error > 0 indicates that D($T_{res}$) underestimates D($T_{res}$ + $R_{uncorr}$) and the residual dose error < 0 indicates that D($T_{res}$) overestimates D($T_{res}$ + $R_{uncorr}$).

The best estimation of the delivered dose in this study is when both residual translation and uncorrected rotation, $T_{res}$ + $R_{uncorr}$ are included in the dose reconstruction, that is, D($T_{res}$ + $R_{uncorr}$) which was compared against the translation‐only estimate of the delivered dose, D($T_{res}$). The residual dose error $\Delta D_{Residual}$ (using Equation (6)) is shown in Figure 5. $\Delta D_{Residual}>$ 0 indicates when D($T_{res}$) underestimates the best estimated delivered dose, D($T_{res}$ + $R_{uncorr}$) and $\Delta D_{Residual}<$ 0 indicates when D($T_{res}$) overestimates the best estimated delivered dose, D($T_{res}$ + $R_{uncorr}$). This demonstrates that uncorrected rotation induces residual dose error (though less than 5% in most of the fractions in this study), resulting in less dose to the CTV and PTV in 43% and 59% treatment fractions, respectively, and more dose to bladder and rectum in 51% and 53% treatment fractions, respectively (Figure 5) compared to translation‐only estimate of the delivered dose.

## 4 DISCUSSION

In this work, we have reconstructed the dynamic delivered dose and determined the dose difference from the planned dose, including residual translation and uncorrected prostate rotation ($T_{res}$ + $R_{uncorr}$) and then including only residual translation ($T_{res}$). We then quantified the magnitude of the residual dose errors due to uncorrected rotation for 158 treatment fractions. Previous studies have investigated the rotation‐induced dose errors by rotating the prostate volume by a constant amount from the original planned position, and hence, the interplay effects between the tumor motion and the machine were neglected.^15^, ^17^, ^20^ In our study, we have fully accounted for these dynamic effects to quantify the dose differences caused by intrafractional rotation. One main assumption in our study was that the PTV, the high‐dose region in the bladder (bladder V6Gy) and rectum (rectum V6Gy) rotate rigidly with CTV around the rotation centroid. This was considered as a first order approximation as the dose gradient is steep so that the high‐dose regions rotate together around the same centroid. We also note that neither the uncertainties in the target definition nor the seed localization error were considered in this study.

From the fractional data analysis in our study (Figure 3), we have found that the best estimated delivered dose in this study and its corresponding dose difference from the plan ΔD($T_{res}$ + $R_{uncorr}$) were larger than 5% in one fraction for PTV D95, in one fraction for bladder V6Gy, and in five fractions for rectum V6Gy out of 158 treatment fractions. The dose differences to CTV D98 were always within 5% of the planned dose in all treatment fractions. While the hypofractionation (> 6 Gy per fraction) delivered with SBRT in four to five fractions has become an alternative treatment strategy showing similar results to conventionally fractioned RT,^34^ whether treatment fractions can be further reduced is an ongoing research matter.^35^, ^36^ The fractional data analysis in our study gives a first order estimation to the motion‐induced dose error due to tumor translational and rotational motion for single fraction treatments.

The cumulative dose, ∑D($T_{res}$ + $R_{uncorr}$) and ∑D($T_{res}$) were always within 5% of the planned dose for all four structures for every patient. This is because the motion‐induced dose errors in a treatment fraction maybe compensated in other fractions, and hence, motion‐induced dose error becomes less of a problem as the number of treatment fractions increases. Therefore, our data suggest that the 3–5 mm CTV–PTV margin will result in minimal dosimetric impact of uncorrected prostate rotation, provided a gating strategy is used for translational motion. However, in this study, the prostate shapes were nearly spherical (the mean and standard deviation of sphericity of the CTV were 0.91 and 0.07, respectively) for all patients, which means that the tumor volume was likely to be insensitive to any rotation; a non‐spherical tumor volume (such as, seminal vesicles included in the CTV) would likely cause larger dosimetric difference.

Though the dose difference due to $T_{res}$ + $R_{uncorr}$ was below 5% for CTV D98 in all treatment fractions, we should note that the inclusion of dynamic rotation, $R_{uncorr}$, in the dose reconstruction induced residual dose error (though small in magnitude) in >40% of the treatment fractions for all four structures (Figure 5). The result of applying the 3 mm/5 s KIM tolerance (5 patients) shows the motion‐induced dose error to increase for both translation only and translation + rotation induced dose as compared to 2 mm/5 s KIM tolerance (27 patients). As both the dose errors (ΔD($T_{res}$ + $R_{uncorr}$) and ΔD($T_{res}$)) increase with a larger treatment tolerance, the residual error due to rotation ($\Delta D_{Residual}$) shown in Figure 5 does not appear to depend on the gating criteria. Correcting this residual dose error will further improve the dosimetric accuracy. A few studies have attempted to correct for this error by applying an equivalent couch rotation, gantry, and collimator rotation and employing multi‐leaf collimator (MLC) tracking. 6 DoF treatment couches exist that could be used for correcting rotation in real‐time; however, they are limited to correcting rotation by only a few degrees.^37^, ^38^ Rotational correction has been demonstrated by collimator angle adjustment in step‐and‐shoot intensity‐modulated radiation therapy plans^15^ and later was extended to VMAT plans.^39^ This technique was applied for rotational correction up to 15° along the LR axis. The same technique was used to correct for roll and pitch error for head and neck cancer patients and found to improve the target coverage.^40^ Electromagnetically guided MLC adaptation to rotational tumor motion was also investigated,^41^, ^42^ and a superior dose distribution was achieved by applying rotational corrections as compared to standard‐of‐care treatment procedure.^41^ Taking into account the patient‐specific geometric variation and replanning can also improve the dosimetric accuracy, however, this process significantly adds extra workload and is time consuming.^43^ To reduce the 6D positional shift into a 3D translational shift, a mathematical formulation was proposed,^44^ which can also be further investigated to correct for rotational error. Another promising option is daily online replanning^45^ on a MR‐Linac or Ethos platform to compensate for the tumor motion variation prior to every treatment fraction. From our data, we see that the rotational motion to be nearly constant throughout some fractions for some patients. For these fractions, an online replanning by compensating the rotation at the beginning of the treatment might help to reduce the treatment margin. However, there were also some fractions in our data, when the magnitude of the rotational motion varied during a single treatment fraction. For these fractions, even if the rotational motion is compensated at the beginning of the treatment with replanning, a margin reduction may end up not providing full dose coverage as the motion varies intrafractionally. Therefore, the tumor motion management and the corresponding margin reduction strategies should be carefully evaluated even when advanced treatment planning methods, such as, daily adaptive online replanning techniques are available.

Future work should focus on investigating the dosimetric impact of rotation for other tumor sites such as lung^46^ and liver,^47^ where significant rotations are observed during treatment and non‐spherical volumes are more common. It will be interesting to study how well the traditionally applied margins provide dose coverage when rotation is included in the dose reconstruction method for these organs. Furthermore, to accurately estimate the delivered dose, instead of using a rigid body assumption, utilization of prostate deformation models will be valuable to replicate the actual patient situation. Another promising option is monitoring and correcting the motion‐induced dose error during treatment and adapting to the dose error in real‐time. While monitoring the geometric error in real‐time provides some measure of the accuracy of the treatment delivery, it is not directly correlated with the dosimetric error. Real‐time motion‐induced dose error monitoring^25^ and its adaptation^48^, ^49^ will be beneficial to eliminate any significant dose error arising due to intrafraction tumor movements.

## 5 CONCLUSIONS

The dosimetric impact of tumor rotation on a large prostate cancer patient cohort was quantified. This study suggests that a 3–5 mm treatment margin provides adequate coverage to account for intrafraction prostate translational and rotational motion, when an appropriate gating threshold is applied to correct for translational motion. Residual dose error due to uncorrected rotation exists which may be corrected with different treatment adaptation techniques to further improve dosimetric accuracy.

## CONFLICT OF INTEREST

Paul J. Keall and Per Rugaard Poulsen are inventors on a patent related to the KIM technology that is licensed to Varian Medical Systems by Stanford University. Authors Paul J. Keall, Doan Trang Nguyen, Ricky O'Brien, and Per Rugaard Poulsen are inventors on additional patents/patent applications related to the KIM technology that have been assigned to the company SeeTreat. Paul J. Keall and Doan Trang Nguyen are founders and directors of SeeTreat. Authors Per Rugaard Poulsen and Thomas Ravkilde have shares in the company Ardos that owns the rights to the DoseTracker technology.
